# Serial Monitoring of Lead aVR in Patients with Prolonged Unconsciousness Following Tricyclic Antidepressant Overdose

**DOI:** 10.4306/pi.2008.5.4.247

**Published:** 2008-12-31

**Authors:** Kyoung Ho Choi, Kyoung-Uk Lee

**Affiliations:** 1 Department of Emergency Medicine, Uijeongbu St. Mary's Hospital, The Catholic University of Korea, College of Medicine, Seoul, Korea.; 2 Department of Psychiatry, Uijeongbu St. Mary's Hospital, The Catholic University of Korea, College of Medicine, Seoul, Korea.

**Keywords:** Tricyclic antidepressant, Overdose, Electrocardiography, Unconsciousness

## Abstract

Severe cardiac and neurologic toxicities of tricyclic antidepressant (TCA) overdose have been reported since the introduction of TCAs in 1950s. Despite the decreased numbers of TCA overdoses, the mortality and morbidity rates of TCA overdose have remained constantly high. Clinical manifestations of TCA overdose are characterized by unconsciousness and specific electrocardiography (ECG) abnormalities such as prolongation of the PR and QTc intervals, widening of the QRS duration, and an increased R wave and R/S ratio in lead aVR. We report a case with unusually prolonged unconsciousness without initial stem reflexes for 7 days and multiple ECG abnormalities following TCA overdose. It is suggested that the serial monitoring of R wave and R/S ratio in lead aVR might be informative in predicting recovery from toxicity following TCA overdose.

## Introduction

Suicide is a significant public health issue. Suicide is generally a complication of a psychiatric disorder. The most common psychiatric conditions associated with suicide or serious suicide attempts are mood disorders.^1^-^3^ Therefore, successful antidepressant treatment is important to prevent suicide. However, drug overdose is one of the most common means of committing suicide.^4^ Thus, the safety of antidepressants following an overdose is critical because of the high risk of suicide attempts in depressed patients. Until recently tricyclic antidepressants (TCAs), which were first introduced in the late 1950s, have been notorious for fatalities following overdose.^5^

Despite a reduction in the total number of poisonings by TCAs, mostly due to the introduction of safer newer antidepressants, the incidence of fatalities due to a TCA overdose has remained constantly high.^5^,^6^ Specific electrocardiography (ECG) abnormalities and unconsciousness characterize TCA overdose.^7^-^10^ We report a case with unusually prolonged unconsciousness without initial stem reflexes and with multiple ECG abnormalities following TCA overdose.

## Case

A forty-five years old male presented to the emergency center with decreased mentality following an overdose of amitriptyline 1.5 g and two benzodiazepines (diazepam 150 mg and lorazepam 15 mg). Five years ago he was diagnosed as suffering from major depressive disorder (MDD) and alcohol dependence and has been treated with a daily dose of amitriptyline 100 mg, diazepam 10 mg and lorazepam 1 mg. His symptoms of depression were aggravated six months ago after his wife left him, and he tried to commit suicide following a physical fight in a drunken state. Thirty-eight hours after ingestion, he was found unconscious by his lover. On arrival at the centre, he was comatose with a Glasgow Coma Scale (GCS) score of 3, all brain stem reflexes were absent, and his pupils were dilated and sluggish. Physical examinations revealed a high blood pressure of 154/79 mmHg, a rapid heart rate of 108 beats/minute, dry oral mucosa, and coarse breathing sounds. He received intubation without additional use of sedatives and was attached to a mechanical ventilator. Urine toxicology screening kits for TCAs and benzodiazepines were positive. Fifty grams charcoal was given via an orogastric tube. An ECG showed a PR interval of 158 msec (normal<200 msec), a prolonged QTc interval of 459 msec (normal<400 msec), a QRS duration (QRSD) of 138 msec (normal<120 msec), and increased amplitudes of R wave (R_aVR_) and R/S ratio (R/S_aVR_) in lead aVR of 3.5 mm (normal<3 mm) and 0.77 (normal<0.7), respectively. Alkalization therapy was initiated using a continuous sodium bicarbonate infusion following bolus injection. On the second day, he had CGS score of 5, but there was a positive corneal reflex. Vital signs were normal except for a heart rate of 110 beats/minute. On the third day, all stem reflexes and robbing eye movements were detected. The ECG showed a PR interval of 206 msec, a prolonged QRSD of 152 msec, a QTc interval of 516 msec, and decreased R_aVR_ and R/S_aVR_ of 3 mm and 0.55, respectively. On the sixth day, the GCS score was 10. ECG showed further decreases in R_aVR_ and R/S_aVR_, 1.5 mm and 0.43, respectively, a still prolonged QTc interval of 497 msec, and a QRSD of 154 msec. Alkalization therapy was stopped because the R_aVR_ and R/S_aVR_ and the level of consciousness were returning to normal. On the seventh day, the patient's level of arousal improved. He was able to follow verbal commands and was able to produce an effective cough. The endotracheal tube was removed after weaning the ventilator. Although the patient complained of multiple somatic symptoms, he strongly denied suicidal ideas. On ninth day, he had recovered medically and was transferred to a local hospital at his family's request.

## Discussion

The use of selective serotonin reuptake inhibitors (SSRIs) and the other new antidepressants has been increasing since their introduction in the late 1980s. This trend has been shown to be correlated with falling suicide rates.^11^,^12^ The decreased suicide rates may be explained by the earlier detection and more effective treatment of depression, and the use of antidepressants which are safer than TCAs. Although the prescriptions for TCAs have declined substantially, both in terms of absolute number and as a proportion of all other antidepressants, there were still a considerable number of patients who were started on TCA treatment.^13^ In addition, TCAs still account for most suicides by antidepressant drug overdose.^1^,^2^

This patient presented at about forty hours following a significant TCA overdose, showed unusually prolonged unconsciousness for seven days and multiple ECG abnormalities. Neurologic toxicities in TCA overdose are known to be the result of pharmacological effects, such as the blockade of monoamine reuptake and anticholinergic action.^5^,^14^ The duration of unconsciousness tends to be short lived, usually no more than 24 hours.^14^

Reports of prolonged unconsciousness without stem reflexes following TCA overdose are rare and are usually limited to cases involving large ingestion, multiple TCAs and coingestion.^14^,^15^ This patient had prolonged unconsciousness without initial stem reflexes for seven days. This is not common, although many cases are reported where the duration of unconsciousness is short. Several factors may have been responsible for the prolonged unconsciousness such as the coingestion of two benzodiazepines, delayed presentation so that the patient was not available in time for decontamination, and consequent significant absorption.^5^,^14^,^15^ No other factors could seem to have affected the duration of coma. There was no additional use of sedatives, and significant medical conditions such as hypoxia and hypotension.

**FIGURE 1:**
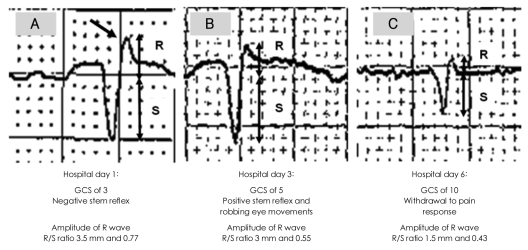
Our patient's ECG at the First, Third, and Sixth hospital day following TCA overdose showed the changes of amplitude of R wave and R/S ratio in lead aVR. In our case, the values of amplitude of R wave and R/S ratio in lead aVR tended to decline in proportion to improvements in GCS scores and his clinical condition. The oblique arrow in figure indicates the prominent and right axis deviated R wave in lead aVR. ECG: electrocardiography, TCA: Tricyclic Antidepressant.

Many researchers have been interested in predicting the severity of TCA overdose using specific ECG findings and neurologic scoring systems.^5^,^7^-^10^,^18^,^19^ Serial ECGs are known to be accurate bedside tests for evaluating the toxicity of TCA overdose because ECG abnormalities usually precede the symptoms of toxicity.^5^,^9^,^10^ Serial estimations of the amplitudes of R wave (R_aVR_) and R/S ratio (R/S_aVR_) in lead aVR are, in particular, very easy to monitor, and provide an accurate method of predicting toxicity with high sensitivity and specificity.^5^,^9^,^17^,^18^ Markedly prolonged QRS duration of more than 160 msec, and R_aVR_ of more than 3 mm and R/S_aVR_ of more than 0.7 have also been reported to predict the high incidence of seizure, progression of toxicity, and fatal outcomes, even if the patients are asymptomatic.^9^,^18^ The present case showed a continuously prolonged PR interval, QRS duration, and QTc interval from initial presentation to the recovery of consciousness. However, R_aVR_ and R/S_aVR_ tended to decline in inverse proportion to the changes in his GCS scores and his clinical condition, as shown in Figure 1. This patient's case suggests that the serial monitoring of R_aVR_ and R/S_aVR_ might be informative in predicting recovery from toxicity following TCA overdose.

Treatments of TCA overdose are mainly divided into the decontamination of drugs and special treatments targeting toxic symptoms.^5^,^19^,^20^ Patients presenting within 12 hours following overdose can receive decontamination because absorption is significantly delayed due to the anticholinergic effect of TCAs. Activated charcoal (AC) should be given via orogastric tube regardless of the arrival times.^5^ Prolonged QRS duration of more than 100 msec, wide QRS tachycardia, R_aVR_ of more than 3 mm and R/S_aVR_ of more than 0.7, and metabolic acidosis are indications of alkalization therapy following TCA overdose. Signs of neurologic toxicity including coma, agitation and delirium, are not indications for alkalization therapy.^5^,^9^,^10^,^14^,^19^,^20^ In the present case, decontamination was not performed, but AC was given via orogastric tube. We started and maintained the alkalization therapy for five days based on ECG abnormalities. We stopped the alkalization therapy at the sixth day after the restoration of a normal R_aVR_ and R/S_aVR_. At the seventh day, our patient regained consciousness. Seizures following TCA overdose are warnings of impending potentially fatal outcomes including hypotension and arrhythmia, but they usually last for only a short time and spontaneously resolve.^5^,^7^,^14^,^19^,^20^ Hypotension in TCA overdose is related to the depletion of neurotransmitters and an alpha-adrenergic antagonistic effect. It is usually resistant to fluid therapy and should be treated with dopamine and norepinephrine.^5^,^9^,^19^

In summary, knowledge about the toxicities of TCAs and management of their toxicities, checking drug compliance, early detection of suicide risk, and effective management of depression may help to reduce fatalities due to drug overdose. Further studies evaluating the usefulness of serial estimations of R_aVR_ and R/S_aVR_ may be necessary.
